# Enhancing the prediction of hospitalization from a COVID-19 agent-based model: A Bayesian method for model parameter estimation

**DOI:** 10.1371/journal.pone.0264704

**Published:** 2022-03-01

**Authors:** Emily Hadley, Sarah Rhea, Kasey Jones, Lei Li, Marie Stoner, Georgiy Bobashev

**Affiliations:** 1 RTI International, Durham, NC, United States of America; 2 Department of Population Health and Pathobiology, North Carolina State University, Raleigh, NC, United States of America; University of Catanzaro: Universita degli Studi Magna Graecia di Catanzaro, ITALY

## Abstract

Agent-based models (ABMs) have become a common tool for estimating demand for hospital beds during the COVID-19 pandemic. A key parameter in these ABMs is the probability of hospitalization for agents with COVID-19. Many published COVID-19 ABMs use either single point or age-specific estimates of the probability of hospitalization for agents with COVID-19, omitting key factors: comorbidities and testing status (i.e., received vs. did not receive COVID-19 test). These omissions can inhibit interpretability, particularly by stakeholders seeking to use an ABM for transparent decision-making. We introduce a straightforward yet novel application of Bayes’ theorem with inputs from aggregated hospital data to better incorporate these factors in an ABM. We update input parameters for a North Carolina COVID-19 ABM using this approach, demonstrate sensitivity to input data selections, and highlight the enhanced interpretability and accuracy of the method and the predictions. We propose that even in tumultuous scenarios with limited information like the early months of the COVID-19 pandemic, straightforward approaches like this one with discrete, attainable inputs can improve ABMs to better support stakeholders.

## Introduction

A significant challenge of the COVID-19 pandemic has been accurately estimating demand for hospital beds. Hospital beds are generally needed by patients with severe COVID-19 to manage symptoms like shortness of breath, confusion, or hemoptysis, or to manage common complications of severe COVID-19 including pneumonia, hypoxemic respiratory failure/acute respiratory distress syndrome, and sepsis [1,2]. Patients with severe COVID-19 can also rapidly deteriorate, requiring prompt admission to an intensive care unit (ICU) [2]. The increase in demand for hospital beds resulting from the COVID-19 pandemic may strain hospital systems that have traditionally used just-in-time supply management to minimize empty beds [3]. Evidence suggests that the pressure of a high volume of patients with severe COVID-19 has a measurable independent impact on in-hospital mortality [4].

Forecasts of hospital bed demand can help health care and public health officials plan for an increased volume of patients, including preparation (e.g., increasing staffing) and acquiring associated resources (e.g., ventilators, personal protective equipment) [5]. Agent-based models (ABMs) have been among the tools used to develop these forecasts. ABMs can simulate the behavior and experiences of a population of synthetic agents, such as becoming infected with SARS-CoV-2, the causative agent of COVID-19, and then hospitalized.

The probability of hospitalization among agents with COVID-19 (i.e., COVID-19 agents) is a key parameter in ABMs used to forecast hospital bed demand. Patient age and comorbidities are known to be strong predictors of the probability of hospitalization among those with COVID-19, although few published COVID-19 ABMs include these attributes [6]. Additionally, few published COVID-19 ABMs consider the difference in probability of hospitalization according to COVID-19 testing status. However, the discrepancy between tested infections (i.e., laboratory-confirmed, reported COVID-19 cases) and untested infections (i.e., undiagnosed, unreported COVID-19 cases) varies greatly over time and place, with U.S. estimates up to 10 times reported cases to infections [7,8]. Although aggregate hospital data can be used to estimate the probability of hospitalization for tested COVID-19 cases, information on untested infections is scarce, potentially resulting in underprediction of a COVID-19 ABM.

In this manuscript, we describe an ABM built to forecast the total demand for ICU beds and non-ICU beds (i.e., regular inpatient hospital beds) in North Carolina (NC) where we initially assigned the same probability of hospitalization for all agents with COVID-19. We discuss a major limitation of this approach: the failure to account for substantial differences in hospitalization rates by age, comorbidities, or COVID-19 testing status. Convincing our stakeholders who sought to use the ABM for transparent decision-making to accept our ABM prediction results was challenging; in particular, it was difficult to justify why all agents should have the same probability of hospitalization when ample evidence contradicted this model assumption. To overcome this limitation, we demonstrate that a straightforward Bayesian method in the estimation of the model parameters can be used, which requires only aggregate data from NC hospitals and the age and comorbidity status of agents in the synthetic population underlying the ABM. We also show that the probability of COVID-19 testing can be incorporated to allow for different hospitalization rates for tested and untested agents. Our modeling results indicated that this approach could enhance the accuracy and interpretability of model predictions and should be a useful technique in ABM development.

## Materials and methods

### Related work

ABMs have been used throughout the COVID-19 pandemic to forecast a variety of outcomes, including demand for non-ICU beds and ICU beds, with varying levels of geographic specificity. Examples include Donker et al. [9], who used data from Italy to provide early insights for Germany; Weissman et al. [5], who developed the CHIME ABM tool for the Philadelphia region; Tuomisto et al. [10], who developed an ABM called REINA to identify destructive pandemic policies for broad geographic areas; Fort et al. [11], who focused specifically on an ABM for hospitals in the New Orleans region; and Truszkowska [12], who applied an ABM to the outbreak in New Rochelle, New York (NY). In these ABMs, agents become infected with SARS-CoV-2 and can be hospitalized for COVID-19. Model outputs, including estimates of the non-ICU and ICU beds needed by the agents for the duration of the ABM, have been used to inform decisions such as increasing hospital bed capacity and purchasing additional personal protective equipment [5].

A key parameter for estimating these outputs is the probability of hospitalization among COVID-19 agents. This probability has a direct impact on the outputs because an increased probability will lead to more hospitalizations. However, Gao and Dong [13] describe how this probability is challenging to derive in practice. If the number of all positive SARS-CoV-2 infections were known, one could calculate the probability of hospitalization as the number of COVID-19-associated hospitalizations among all SARS-CoV-2 infections. However, the true number of SARS-CoV-2 infections is unknown; in July 2020, CDC estimated that there were 10 times as many SARS-CoV-2 infections as laboratory-confirmed and reported COVID-19 cases [7] An approximate hospitalization rate can be calculated as the number of persons hospitalized with COVID-19 compared to the total number of reported COVID-19 cases, but this number is likely a substantial overestimate of likelihood of hospitalization if used to estimate the probability of hospitalization for all persons with COVID-19. Although Gao and Dong [13] discuss how testing may impact the probability of hospitalization, the ABMs reviewed for this analysis did not specifically use the probability of COVID-19 testing in determining the probability of hospitalization associated with COVID-19.

The literature also strongly suggests that not all persons infected with SARS-CoV-2 have the same probability of hospitalization with COVID-19; specifically, older individuals and individuals with comorbidities (e.g., chronic obstructive pulmonary disease, obesity) appear to have higher likelihoods of requiring hospitalization [6]. Attributes like age and comorbidities can be assigned to agents in ABMs [14]. The parameter for probability of hospitalization can be conditionally assigned for each COVID-19 agent according to the agent’s age and comorbidity status. Kashyap et al. [15] suggested that models that do not use patient age distribution are more likely to overestimate the resource burden of COVID-19.

When specifying the parameter for the probability of hospitalization, several ABMs used the same probability for all agents with COVID-19, regardless of age or comorbidities. Based on data collected at Ochsner Health hospitals in New Orleans in March and early April 2020, Fort et al. [11] used a 9% hospitalization rate. Weismann et al. [5] used internal March 2020 data from Pennsylvania to estimate a hospitalization rate of 2.5% (CI 1%– 5.1%). Donker et al. [9] calculated the hospital admission rate as the cumulative number of hospital admissions divided by the cumulative number of confirmed cases, although no numeric estimate was provided. Hoertel et al. [16] forecasted ICU occupancy and estimated it as four times the mortality rate observed in the ICU.

In March 2020, Verity et al. [17] released age-specific probabilities of hospitalization given SARS-CoV-2 infection. In the same month, Ferguson et al. [18] updated these probabilities to account for non-uniform attack rates by age. When applied to the UK population, these estimates suggested an overall hospitalization rate of 4.4%. Uncertainty estimates were not provided. Many subsequent ABMs throughout 2020 and into 2021 used the direct estimates or modifications of the estimates by Verity et al. [17] or Ferguson et al. [18] as shown in Table 1, including Tuomisto et al. [10], Silva et al. [19], Kerr et al. [20], and Truszkowska et al. [12]. Tuomisto et al. [10], Silva et al [19]. and Kerr et al. [20] models were location-agnostic, while Truszkowska et al. [12] explicitly modeled the small town of New Rochelle, NY. Many models recognized that importance of incorporating age-specific parameters, but also assumed applicability across geographies which may not reflect regional realities.

**Table 1:** Age-specific probabilities of hospitalization given COVID-19 infection.

Age Group	Verity et al. [17] and Tuomisto [10]	Kerr et al. [20] adapted from Verity et al. [17]	Truszkowska et al. [12], Silva et al. [19], & Ferguson et al. [18] adapted from Verity et al. [17]
0 to 9	0	0	0.001
10 to 19	0.001	0.0004	0.003
20 to 29	0.021	0.011	0.012
30 to 39	0.067	0.034	0.032
40 to 49	0.085	0.043	0.049
50 to 59	0.163	0.082	0.102
60 to 69	0.236	0.118	0.166
70 to 79	0.332	0.166	0.243
80 plus	0.368	0.184	0.273

Fewer models explicitly incorporated comorbidities into the probability of hospitalization. Gao and Dong [13] explored which comorbidities were most highly related to the probability of hospitalization and Qian et al. [21] estimated the profile of comorbidities among those hospitalized. Only Kerr et al. [20] explicitly used the comorbidities of each agent to modify the probability of developing severe COVID-19 symptoms that would require hospitalization.

The models that considered age- or comorbidity-specific probabilities of hospitalization generally relied on conditional probabilities alone. Gao and Dong [13,22] extended the use of conditional probabilities by proposing a Bayesian framework with which to estimate the probability of hospitalization.

Throughout the COVID-19 pandemic, stakeholders including government officials have utilized agent-based models for key policy decisions [23]. The re-use of age estimates and the lack of use of comorbidity or testing attributes estimates in many of these models reflect the lack of available information in the early months of the COVID-19 pandemic as well as a need by stakeholders for rapid model development. However, these omissions conflict with a stakeholder demand for interpretability to facilitate transparent decisions and the reuse of parameters in different geographies may affect model reliability. A lack of interpretability and reliability of COVID-19 ABMs have contributed to critiques of their use for policymaking [24].

### ABM design

We developed an ABM in Python to generate 30-day forecasts for the demand for ICU and non-ICU beds for all agents (both with and without COVID-19) in North Carolina [25]. Estimates were provided at the state level and for the 10 Local Health Director (LHD) regions in North Carolina from March 2020 through December 2020. The ABM modeled agent movement between the community, short term care facilities, long-term acute-care hospitals (LTACHs), and nursing homes. The ABM utilized all agents from NC in the RTI SynthPop [26]. The RTI SynthPop is a virtual, anonymized synthetic population that is representative of real people and can be used for simulation efforts like this one. Each person in the synthetic population is called an agent, and agent attributes included age and a binary flag for presence of comorbidities that was previously developed for an ABM of *Clostridioides difficile* Infection [14]. Note that one limitation of the comorbidity assignments is that they were only estimated for three age ranges (0–49, 50–64, and 65+ years), with no agents under 50 being assigned comorbidities [14]. The ABM utilized a susceptible-exposed-infected-recovered (SEIR) model for SARS-CoV-2 with different probabilities of infection by age, and agents could be hospitalized with COVID-19 during the “Infected” stage.

When determining the probability of hospitalization with COVID-19, we initially used a single point estimate for all infected agents similar to ABMs developed by Fort et al. [11], Weismann et al. [5], Donker et al. [9], and Hoertel et al. [16]. This value was set to 2.2%. This was half of the value estimated from Ferguson et al. [18] and was equivalent to the CDC estimate of 4.6 hospitalizations per 100,000 persons [27]. However, literature contradicted the assumption that all infected agents should have the same probability of hospitalization with COVID-19.

We started with age and comorbidity status, which were available in the underlying synthetic population, as well as testing status which was estimated in the ABM. A testing status of “tested” meant that a person had received any type of test for COVID-19, while a testing status of “untested” meant that a person had not received any type of test for COVID-19. To incorporate these attributes in the probability of hospitalization with COVID-19, we proposed a Bayesian framework. While Gao and Dong focus on approximating a Bayesian posterior with beta distributions, we used a straightforward application of the Bayes’ theorem. We aimed to use local NC hospital data to capture the conditional relationships between age, comorbidities, and hospitalization from symptoms of COVID-19 while also accounting for unreported SARS-CoV-2 infections. With this method, we were able to use NC-specific data rather than relying on estimates derived from different populations that may not reflect NC.

### Bayesian specification

Our proposed method was intentionally designed to require few inputs that were reasonably accessible given the pandemic and to be interpretable for stakeholders. A similar method had been used by Gao and Dong [13,22]. To estimate the conditional probability of hospitalization associated with COVID-19 *p(hosp)* given an agent’s age and comorbidity status (i.e., presence or absence of comorbidities), we use the following formula for Bayes’ theorem:

$$p(hosp|age\&comorbidities)=\frac{p(age\&comorbidities|hosp)*p(hosp)}{p(age\&comorbidities)}$$

The same equation can be used separately to obtain estimates for tested infections (i.e., laboratory confirmed and reported COVID-19 cases) and untested infections. It is important to note that “untested” is not an official status in the ABM but is understood to mean “untested prior to hospitalization” because it is likely that patients presenting with possible COVID-19 at a hospital would be tested upon admission. This does not impact the model because the probability of hospitalization is what the ABM uses to identify agents for hospital admission, so subsequent testing at the hospital would not impact this parameter. At the time that the model was initially constructed in June 2020, it was estimated that 10% of SARS-CoV-2 infections were reported as COVID-19 cases [7].

Table 2 highlights differences in the methods used to calculate each component of the Bayesian equation between tested and untested infections. One key difference is that the probability of hospitalization for agents for COVID-19 agents is expected to be higher among tested infections than untested infections. This is based on a comparison with the CDC overall estimate of probability of hospitalization, and the suggested estimate that untested infections are larger in number than tested infections, particularly in the early months of the pandemic in 2020 when testing was limited.

**Table 2:** Methods for calculating portions of Bayesian equation by COVID-19 testing status.

	Tested and Reported Cases	Untested Infections
*p(age & comorbidities | hosp)*	Calculated using crosstabs of age & comorbidity status among hospitalized with COVD-19 provided by NC DHHS	Assumed to be the same as for Tested and Reported Cases
*p(hosp)*	Estimate of 8.5% in June 2020 provided by NC DHHS	Estimated at 1.2% from expert input to calibrate with ~1.9% overall hospitalization
*p(age & comorbidities)*	Calculated from crosstab age and comorbidities among those positive with COVID-19 and tested in synthetic population	Calculated from crosstab of age and comorbidities among those positive with COVID-19 and untested in synthetic population

NC DHHS: North Carolina Department of Health and Human Services.

Another key difference is the joint probability of age and comorbidity status. These estimates were calculated from those agents estimated to have COVID-19 in the ABM. The ABM included a separate calculation of probability of SARS-CoV-2 infection through a SEIR model that accounted for differences in likelihood of infection by age.

### Data considerations

One important advantage of our method is that it requires few data inputs for ease of calculation and interpretation. We obtained the following aggregated data quickly from the North Carolina Department of Health and Human Services (NC DHHS) which was crucial in a setting where access to data was limited, and record-level patient data would have been extremely difficult to access:

- Count of hospitalizations disaggregated by age group and COVID-19 status
- Count of hospitalizations disaggregated by comorbidities and COVID-19 status
- Count of COVID-19 tests by result and by age group

The results from the Bayesian calculation were used directly in the ABM. Each agent with COVID-19 on a given day received a probability of hospitalization given their age and comorbidity status and was assigned a hospitalization status based on this probability. Disease progression including admission to the ICU was handled in the ABM itself.

Non-public, deidentified data were obtained and fully anonymized prior to access. The RTI IRB reviewed this study and determined that informed consent was not required as it does not constitute research with human subjects.

## Results

The model parameters obtained by using our Bayesian method are presented in Table 3. These values were calculated using hospitalization data from June 2020. These exact values should be interpreted as the probability of hospitalization for agents in the NC synthetic population used in the ABM, which has a slightly smaller total population than NC’s current population. The results should not be extrapolated to represent the current NC population. The overall hospitalization rate was 1.93%.

**Table 3:** ABM probability of hospitalization from COVID-19 from Bayes calculations.

	Tested and Reported COVID-19 Case		Untested COVID-19 Infection	
Age Range	No Comorbidities	Comorbidities	No Comorbidities	Comorbidities
0 to 49	0.037	NA	0.008	NA
50 to 64	0.035	0.461	0.007	0.098
65+	0.121	0.411	0.026	0.087

The model parameters in Table 3 were used in our ABM run. As a comparison, we also ran our ABM using a single point estimate of the overall hospitalization rate of 0.0193. The frequency distributions of the NC synthetic population by age group and comorbidity status were summarized in Table 4. When the overall hospitalization rate was used, among agents of the age group 65+ 12.0% were hospitalized with COVID-19, and among agents with comorbidities 11.1% were hospitalized with COVID-19. When the hospitalization rates obtained by using our Bayesian method were used, among agents of the age group 65+ 39.8% were hospitalized with COVID-19, and among agents with comorbidities 56.5% were hospitalized with COVID-19. The average age of hospitalized agents was 37.6 years for the single point estimate method but 57.4 years for the Bayes method.

**Table 4:** Percentage of agents hospitalized with COVID-19 by subgroup in NC synthetic population when an overall hospitalization rate (single point estimate) or age group and comorbidity-specific hospitalization rates (Bayesian method) were used.

	Age Range	Single Point Estimate (0.0193)	Bayes’ Method
With Comorbidities	0 to 49	NA*	NA*
	50 to 64	4.5%	24.6%
	65+	6.6%	31.9%
Without Comorbidities	0 to 49	69.0%	29.8%
	50 to 64	14.5%	5.9%
	65+	5.4%	7.7%
Total (Hospitalized with COVID-19)		100%	100%

*Comorbidity status is not assigned to agents in the 0 to 49 age range in the NC synthetic population.

**Fig 1:**
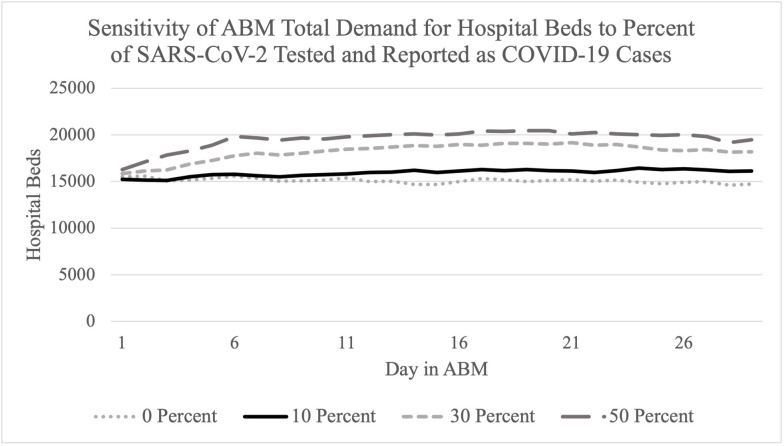
Sensitivity analysis of ABM results of total demand for hospital beds to the parameter of percentage of SARS-CoV-2 infections tested and reported as COVID-19 cases.

A critical parameter choice made to obtain the results in Table 3 was the assumption that 10% of positive SAS-CoV-2 infections were tested and reported as COVID-19 cases. This parameter estimate was made using available literature but impossible to know with certainty. Fig 1 highlights a sensitivity analysis of ABM results for a 30-day forecast of the total demand for hospital beds needed by all agents (with and without COVID-19) under scenarios with differing percentages of tested SARS-CoV-2 infections reported as COVID-19 cases. This hypothetical scenario is used only for the purposes of sensitivity testing and assumes a low effective R range (1–1.2). The assumed value of 10% most closely approximates the likely expected scenario with a low effective R range of a small increase in demand for hospital beds over a 30-day period. The larger parameters of 30% and 50% of positive SARS-CoV-2 infections tested and reported as COVID-19 cases are associated with larger increases in hospital bed demand that may be larger than expected for a low effective R range.

## Discussion

We described an application of Bayes theorem that enhanced the prediction of hospitalization with COVID-19 in an ABM and required few data inputs. Our modeling results showed that this Bayesian approach yielded more accurate predictions that better aligned with observations in the literature that older persons and persons with comorbidities are more likely to be hospitalized with COVID-19. This approach also can lead to improvements in other ABM outcomes of interests that could use accurate age and comorbidity status for hospitalized agents such as length of hospital stay, need for a ventilator, or disease progression. Our Bayesian approach enabled us to provide more interpretable results to stakeholders who often questioned the explainability of the single point estimate method and preferred more detailed prediction results that could facilitate transparent decision-making.

In general, our results suggest that the conditional probability of hospitalization for those with comorbidities is higher than those without comorbidities and potentially substantially higher than the solely age-specific values used by other ABMs. Conversely, estimates for those without comorbidities, even in higher age groups, may be lower than solely age-specific estimates. Notably, the probability of hospitalization for untested infections with comorbidities was suggested to be higher than the probability of hospitalization for tested infections without comorbidities in the case of adults ages 50 to 64 years. These results again highlight the additional accuracy and interpretability of ABM results from using Bayes’ theorem.

Our sensitivity analysis indicated that this approach can be sensitive to the percentage of COVID-19 infections that are tested and reported as cases. Available literature should be used to select this parameter and be informed by expert input as appropriate.

The advantages of our proposed approach include allowing for use of additional details such as age, comorbidity status, and testing status when calculating the probability of hospitalization with COVID-19. It is flexible and can be updated when new data or updated expert opinions are available. It enhances the geographic granularity of an ABM by more accurately reflecting the probability of hospitalization given characteristics that are geographically represented in the ABM, and thus likely yielding more accurate regional and local estimates.

The practical applications of this approach can be extended frommodeling hospital bed capacity to modeling other healthcare outcomes. A more accurate reflection of the age and comorbidity characteristics of the patient population could be useful in models related to improving the efficiency of healthcare systems [28] by supporting more granular information related to patient needs for rehabilitation, nursing home, and long-term care facilities. Similarly, information on these characteristics could also impact models related to the quality of work experience for healthcare workers [29] by helping anticipate the broader age- and comorbidity-specific needs of the patient population and preparing staff skills to align with those needs.

This approach is also applicable to other diseases and epidemics. Bayes’ theorem required few data inputs, and the necessary inputs were discrete, aggregated, and often accessible from local or regional public health agencies. Its implementation is simple and straightforward. It is well-suited to a tumultuous scenario with limited information such as the early months of the COVID-19 pandemic and when prediction results with greater accuracy and interpretability are desired.

### Limitations

A variety of limitations exist for this work. First, as discussed in Section 4, the specific Bayesian probabilities in this analysis should only be interpreted in the context of the NC synthetic population used in this project. Comorbidity status is only assigned to adults aged 50 and older in the synthetic population, and the comorbidity status assignment was based on health care-associated risk, which is slightly different than the one published by CDC for COVID-19 hospitalization [6,14]. A second limitation is that in this initial analysis, separate probabilities of age given hospitalization with COVID-19 and comorbidity status given hospitalization with COVID-19 were provided by NC DHHS. The calculation of the joint probability of age and comorbidity status given hospitalization with COVID-19 assumed that the underlying probabilities were independent, an assumption that may not be true. In later versions of the ABM, we subsequently made updates to this calculation and used data that included the joint distribution of age and comorbidity status given hospitalization with COVID-19. A third limitation is that the calculations did not account for differences in specificity and sensitivity for different types of COVID-19 tests and may overestimate the quality of testing results. Finally, the assumption was made that although age impacted the likelihood of infection with COVID-19, neither age nor comorbidity status influenced the likelihood of receiving a COVID-19 test once infected with SARS-CoV-2.

## Conclusion

We used Bayes’ theorem to estimate the probability of hospitalization with COVID-19, given age, comorbidity, and testing status of agents with COVID-19 in a NC ABM forecasting demand for non-ICU and ICU beds. We demonstrated that the analysis was sensitive to the percentage of infections that are tested and reported as cases and the selection of the likelihood of hospitalization for untested infections. This straightforward yet innovative approach enhanced accuracy and interpretability of model predictions for stakeholders seeking transparent decision-making tools and may be useful for scenarios with limited information similar to the early months of the COVID-19 pandemic.

The findings and conclusions in this publication are those of the authors and do not necessarily represent the views of the North Carolina Department of Health and Human Services, Division of Public Health or the Centers for Disease Control and Prevention (CDC).

## Supporting information

S1 Table Data used to create Fig 1. (XLSX)
